# Characterization of *Enterococcus* Isolates Colonizing the Intestinal Tract of Intensive Care Unit Patients Receiving Selective Digestive Decontamination

**DOI:** 10.3389/fmicb.2017.01596

**Published:** 2017-08-28

**Authors:** Teresita d. J. Bello Gonzalez, Phu Pham, Janetta Top, Rob J. L. Willems, Willem van Schaik, Mark W. J. van Passel, Hauke Smidt

**Affiliations:** ^1^Laboratory of Microbiology, Wageningen University & Research Wageningen, Netherlands; ^2^Department of Medical Microbiology, University Medical Center Utrecht Utrecht, Netherlands; ^3^Centre for Zoonoses and Environmental Microbiology, National Institute for Public Health and the Environment Bilthoven, Netherlands

**Keywords:** *Enterococcus*, intestinal colonization, antibiotic resistance, virulence factors, antibiotic prophylactic therapy, selective digestive decontamination

## Abstract

Enterococci have emerged as important opportunistic pathogens in intensive care units (ICUs). In this study, enterococcal population size and *Enterococcus* isolates colonizing the intestinal tract of ICU patients receiving Selective Digestive Decontamination (SDD) were investigated. All nine patients included in the study showed substantial shifts in the enterococcal 16S rRNA gene copy number in the gut microbiota during the hospitalization period. Furthermore, 41 *Enterococcus* spp. strains were isolated and characterized from these patients at different time points during and after ICU hospitalization, including *E. faecalis* (*n* = 13), *E. faecium* (*n* = 23), and five isolates that could not unequivocally assigned to a specific species (*E. sp. n* = 5) Multi locus sequence typing revealed a high prevalence of ST 6 in *E. faecalis* isolates (46%) and ST 117 in *E. faecium* (52%). Furthermore, antibiotic resistance phenotypes, including macrolide and vancomycin resistance, as well as virulence factor-encoding genes [*asa1, esp*-*fm, esp*-*fs, hyl*, and *cyl* (B)] were investigated in all isolates. Resistance to ampicillin and tetracycline was observed in 25 (61%) and 19 (46%) isolates, respectively. Furthermore, 30 out of 41 isolates harbored the *erm* (B) gene, mainly present in *E. faecium* isolates (78%). The most prevalent virulence genes were *asa*1 in *E. faecalis* (54%) and *esp* (*esp-fm*, 74%; *esp-fs*, 39%). Six out of nine patients developed nosocomial enterococcal infections, however, corresponding clinical isolates were unfortunately not available for further analysis. Our results show that multiple *Enterococcus* species, carrying several antibiotic resistance and virulence genes, occurred simultaneously in patients receiving SDD therapy, with varying prevalence dynamics over time. Furthermore, simultaneous presence and/or replacement of *E. faecium* STs was observed-, reinforcing the importance of screening multiple isolates to comprehensively characterize enterococcal diversity in ICU patients.

## Introduction

The genus *Enterococcus* encompasses indigenous commensal bacteria reported from the human and animal gut as well as the oral cavity and vagina in humans, where they have adapted to nutrient-rich, oxygen-depleted, and ecologically complex environments (Fisher and Phillips, 2009).

In the human gut, the genus *Enterococcus* can constitute up to 1% of the total bacterial microbiota in healthy individuals, with *Enterococcus faecium* and *Enterococcus faecalis* as most common species (Sghir et al., 2000). In contrast to their commensal role, over the past decades *E. faecium* and *E. faecalis* have also emerged as agents of nosocomial infections such as endocarditis, bacteraemia, meningitis, wound, and urinary tract infections (Guzman Prieto et al., 2016). In addition, other enterococcal species including *Enterococcus durans, Enterococcus avium, Enterococcus gallinarum, Enterococcus casseliflavus, Enterococcus raffinosus*, and *Enterococcus hirae* have sporadically been associated with infections in humans (Klein, 2003).

Most of the *E. faecium* and *E. faecalis* infections are opportunistic and are increasingly difficult to treat due to high rates of resistance to β-lactams, aminoglycosides, and vancomycin, which are mostly associated with *E. faecium* strains (Cattaneo et al., 2010). Similar to resistance genes, virulence genes are also frequently encoded on mobile elements and are therefore thought to disseminate frequently via intra- and interspecies horizontal gene transfer within the genus *Enterococcus* (Laverde Gomez et al., 2011). Both *E. faecium* and *E. faecalis* can carry a variety of genes that contribute to virulence in the immunocompromised patient. For *E. faecalis* these include genes encoding aggregation substance (*asa1*) (Galli and Wirth, 1991), cytolysin (*cyl*) (Jett et al., 1992), and enterococcal surface protein (*esp-fs*) (Vankerckhoven et al., 2004),whereas for *E. faecium* genes associated with virulence encode a putative hyalorunidase (*hyl*) (Klare et al., 2005) and enterococcal surface protein (*esp-fm*) (Hendrickx et al., 2013), among others.

Studies using Multi Locus Sequence Typing (MLST) have shown that there is a remarkable difference in the population structure between *E. faecalis* and *E. faecium* (Palmer et al., 2014). The phylogeny of *E. faecalis* did not reveal clustering of strains according to their source, e.g., human, clinical, or animal strains (Guzman Prieto et al., 2016).

In contrast, in *E. faecium*, high-risk clonal-complexes exist, which exhibit high levels of antibiotic resistance and are significantly associated with clinical infections in hospitalized patients (Leavis et al., 2006; Willems et al., 2012). Genome sequencing confirmed that the majority of clinical isolates form a distinct sub-population of *E. faecium* (Lebreton et al., 2013). Recently, Tedim et al. (2015) studied the population biology of intestinal *Enterococcus* isolates from hospitalized and non-hospitalized individuals in different age groups. They found that *E. faecium* populations differ with respect to the observed clonal lineages between hospitalized patients and community-based individuals.

Moreover, the previous identification by whole genome sequencing of *E. faecium* isolates of distinct hospital (A1) and commensal (B) clades suggests that a distinct evolutionary background exists between commensal and clinical isolates (Palmer et al., 2012). Likewise, Muruzábal-Lecumberri et al. (2015) reported a high prevalence of *E. faecalis* sequence type (ST) 6 (CC2) in patients undergoing selective decontamination of the digestive tract and indicated that a rapid detection is necessary to avoid a dissemination outside intensive care units (ICUs).

Patients in an ICU are at a high risk for developing nosocomial infections with multi-drug resistant bacteria and are often exposed to strong selective antibiotic pressure (Streit et al., 2004). Several studies have shown that the exposure of patients to broad-spectrum antibiotics, combined with prolonged hospital stay, can result in colonization by multi-drug resistant enterococci leading to nosocomial transmission and infection (Austin et al., 1999; Carmeli et al., 2002). The prophylactic therapy Selective Digestive Decontamination (SDD) aims to prevent secondary infection with opportunistic pathogens, including Enterobacteriaceae, *Staphylococcus aureus* and yeasts, in ICU patients and to decrease mortality (de Smet et al., 2009).

Previous studies have shown that SDD therapy can select for intestinal colonization by enterococci (Humphreys et al., 1992; de Smet et al., 2009; Benus et al., 2010). A recent meta-analysis of van der Bij et al. (2016), determined the antibiotic resistance rate of Gram-positive cocci in blood and respiratory specimens in 42 Dutch ICUs in the period from 2008 to 2013, indicating that prophylactic therapy was not associated with an increase of antibiotic resistance in Gram-positive cocci. In this study, we characterized *Enterococcus* isolates colonizing the intestinal tract of ICU patients receiving SDD therapy and to evaluate in more detail the genetic relatedness of *E. faecalis* and *E. faecium* isolates, using MLST and Bayesian analysis of the population structure (BAPS). Furthermore, we determined carriage of genes encoding antimicrobial resistance and virulence determinants in this population.

## Materials and methods

### Selection of patients

The patients were selected as part of an approved study to determine the effects of antibiotic prophylactic therapy on antibiotic resistance (Buelow et al., 2014). The inclusion criteria included patients discharged from the ICU who received SDD for at least 96 h. Exclusion criteria included a hospital stay and/or antibiotic treatment prior to ICU admission and discontinuation of SDD before ICU discharge. All patients included in this study were > 18 years of age. The SDD protocol was reviewed and approved by the institutional review board of the University Medical Center Utrecht (Utrecht, The Netherlands). The SDD protocol comprised the oral application of 0.5 g of a paste containing 2% tobramycin 2% polymyxin E and 2% amphotericin B, given four times daily. In addition, a 10 ml suspension containing 80 mg tobramycin, 100 mg polymyxin E and 500 mg amphotericin B was administered through a gastric tube four times daily, and cefotaxime (4 × 1,000 mg) was given intravenously for the first 4 days after ICU admission.

### Samples collection

Fecal samples were collected at different time points during ICU hospitalization and, for five patients, after ICU discharge and cessation of SDD. All the samples were de-identified, received a sample code, and were subsequently categorized according to the collection time for subsequent analyses: ICU stay (including samples collected during ICU hospitalization for up to 40 days, *n* = 27), and post-ICU (samples taken after ICU discharge and discontinuation of SDD, *n* = 7) (Table 1). Samples were collected upon defecation and stored at 4°C for 30 min to 4 h. Two aliquot of 0.5 g of fecal material were collected, one aliquot was stored directly −80°C for fecal DNA isolation, and the other aliquot was suspended in 5 ml of 20 mM anoxic phosphate buffer (pH 7.0) with 40% glycerol, and transferred to −80°C for further analysis.

**Table 1:** Overview of the *Enterococcus* species isolated and characterized per patient during the different time points (ICU stay and Post-ICU).

**Patient ID**	Original patient ID^*^	**Sample collected (days)**	**Location of patient**	**Identification**	**MLST**	**MIC Van (μg/ml)**	**Resistance gene**	**Virulence factor**
Patient 1	120	3	ICU	*E. faecalis*	589	2	N.A.^**^	N.D.^***^
		3	ICU	*E. faecalis*	589	2	*ermB*	N.D
		21	ICU	*E. faecalis*	589	2	N.D	N.D
		41	Post-ICU	*E. faecium*	78	2	N.D	*esp*
		41	Post-ICU	*E. faecium*	78	2	*ermB*	*esp*
		41	Post-ICU	*E. faecalis*	589	2	N.D	N.D
Patient 2	105	17	Post-ICU	*E.sp_1*		0.5	*ermB*	N.D
		17	Post-ICU	*E.sp_2*		0.5	*ermB*	N.D
		17	Post-ICU	*E.sp_3*		16	*ermB, vanC1*	*esp, hyl*
Patient 3	108	2	ICU	*E. faecium*	117	0.5	*ermB*	*esp*
		12	ICU	*E. faecium*	117	0.5	*ermB*	*esp*
		27	Post-ICU	*E. faecalis*	81	0.5	*ermB*	*asa, esp*
Patient 4	157	6	ICU	*E. faecium*	117	1	*ermB*	*esp*
		7	ICU	*E. faecium*	730	1	*ermB*	*esp*
		15	ICU	*E. faecium*	117	1	*ermB*	*esp*
		24	ICU	*E. faecium*	730	1	*ermB*	*esp*
		40	ICU	*E. faecium*	730	1	*ermB*	*esp,asa*
		40	ICU	*E. faecalis*	6	1	*ermB*	*esp,asa*
		40	ICU	*E. faecalis*	6	1	*ermB*	*esp*
		40	ICU	*E. faecalis*	6	1	N.D	*esp*
Patient 5	179	3	ICU	*E. faecium*	117	0.75	*ermB*	*esp*
		3	ICU	*E. faecium*	117	0.75	*ermB*	*esp*
		16	Post-ICU	*E. faecium*	117	0.75	*ermB*	*esp*
		18	Post-ICU	*E. faecium*	117	0.75	*ermB*	*esp*
		16	Post-ICU	*E.sp_4*		0.5	*ermB*	N.D
		18	Post-ICU	*E.sp_5*		0.5	N.A	*esp, asa*
Patient 6	165	4	ICU	*E. faecalis*	16	1	*ermB*	*esp,asa*
		16	ICU	*E. faecalis*	16	1.5	*ermB*	*esp, asa*
Patient 7	180	5	ICU	*E. faecium*	60	0.5	N.A	N.D
		5	ICU	*E. faecium*	117	1	*ermB*	*esp*
		16	ICU	*E. faecium*	361	1	N.D	N.D
Patient 8	169	6	ICU	*E. faecalis*	6	1	*ermB*	*asa*
		6	ICU	*E. faecalis*	6	1	*ermB*	*asa,esp*
		11	ICU	*E. faecalis*	6	1	*ermB*	*asa*
		11	ICU	*E. faecium*	117	1	*ermB*	*esp*
		11	ICU	*E. faecium*	117	1	*ermB*	*esp,asa*
		25	Post-ICU	*E. faecium*	17	1	N.D	N.D
		25	Post-ICU	*E. faecium*	17	1	N.D	N.D
		25	Post-ICU	*E. faecium*	17	1	*ermB*	N.D
Patient 9	163	4	ICU	*E. faecium*	117	0.75	N.D	N.D
		4	ICU	*E. faecium*	730	0.75	*ermB*	*esp*

* *The original code represents the patient assigned number used in previous publications (Buelow et al., 2014, 2017)*.

** *NA, no applicable*.

*** *ND, not detected*.

### Bacterial culture conditions and initial characterization

Enterococci were isolated on Bile-Esculin Agar (BEA) (Oxoid B.V., Landsmeer, The Netherlands). Colonies growing on BEA media were selected based on colony morphology (up to five colonies per plate) for phenotypic characterization (Winn et al., 2006). Haemolysis was determined by cultivation on Blood Agar supplemented with 5% sheep blood (Oxoid) after incubation at 37°C for 24 h. Isolates that showed phenotypic differences in antibiotic resistance patterns (see below) were selected for further characterization.

DNA isolation was performed using the protocol for Gram-positive bacteria of the QIAamp® DNA Mini Kit (Qiagen Benelux B.V., Venlo, The Netherlands). DNA was used for the identification of the isolates and detection of antibiotic resistance and virulence genes by Polymerase Chain Reaction (PCR) as described below. In addition, total bacterial DNA extraction was performed from 0.5 g of fecal material using the modified repeated bead beating method previously described (Salonen et al., 2010) and used for the quantification of the enterococcal population as described below.

### Identification and classification of isolates

The complete bacterial 16S ribosomal RNA (rRNA) gene was amplified from genomic DNA using T7prom-Bact-27-F and Uni-1492-R primers as described previously (Rajilić-Stojanović et al., 2009). The amplified fragments were selected for partial sequence analysis of the 16S rRNA gene (~800 bp) using the 16S-1392R primer 5′- ACGGGCGGTGTGTRC -3′ (GATC Biotech, Cologne, Germany).

Partial 16S rRNA gene sequences of isolates assigned to other *Enterococcus* species obtained in this study were deposited at GenBank under accession numbers KX577731, KX577732, KX577733, KX577734.

### Quantification of the enterococcal population

The enterococcal population present in the fecal material was quantified by qPCR with 16S rRNA gene targeted enterococci-specific primers described by Matsuda et al. (2009). A standard curve was made from bacterial genomic DNA (*E. faecium* E5), using 10-fold dilution series (1.0 × 10^1^–1.0 × 10^9^ DNA copies μl^−1^). The standard curve had a correlation coefficient of *R* = 0.97–0.99 and an efficiency of amplification between 97.2 and 103.1%.

The qPCR was performed with the SYBR-Green PCR kit (Roche Applied Science). The total reaction volume was 10 μl: 5 μl SYBR-Green I, 0.2 μl of each forward and reverse primers, 1.6 μl of sterilized nuclease free water and 3 μl extracted DNA (1 ng/μl). The amplification program included an initial denaturation step at 94°C for 5 min, followed by 40 cycles of denaturation for 20 s at 94°C, annealing for 20 s at 60°C and extension for 50 s at 72^0^C. A melting curve analysis was performed by slowly heating the sample from 60 to 95°C (0.5°C per 15 s) with simultaneous monitoring of fluorescence. All reactions were performed in duplicates in a 384- well PCR plate sealed with optical sealing tape (Bio-Rad) on a iQ5 real-time PCR detection system.

One way-ANOVA was used for statistical analysis to indicate significant differences of enterococcal abundance during and after SDD therapy, with Bonferoni correction (*p* < 0.05) for multiple testing.

### Antimicrobial susceptibility

Vancomycin resistance of enterococci was screened on Mueller-Hinton Agar (MHA) (Oxoid) containing 6 μg/ml vancomycin. Colonies growing on this medium were tested by E-test (Biomerieux) to determine the minimal inhibitory concentration (MIC) of vancomycin, following CLSI guidelines (CLSI, 2013). In addition, the disk diffusion method was used to test for susceptibility to ampicillin (10 μg) and tetracycline (30 μg) (CLSI, 2013).

### Detection of antibiotic resistance- and virulence factor-encoding genes

Antibiotic resistance genes were detected using a multiplex PCR for the vancomycin-resistance genes *van* (A), *van* (B), and *van* (C) (*van* (C1)–*van* (C2)/*van* (*C3*)) (Depardieu et al., 2004), and a single PCR for *erm* (A), *erm* (B), *erm* (C), and *mef* (A)/*mef* (E) genes (Zou et al., 2011). PCR products of *mef* (A) and *mef* (E) genes were discriminated by BamHI restriction analysis, as only *mef* (A) carries a single restriction site, giving rise to fragments of 284 and 64 bp as described previously (Klaassen and Moutin, 2005).

Genes coding for virulence factors, i.e., enterococcal surface protein (*esp-fm, esp-fs*), aggregation substance (*asa1*), cytolysin (*cyl* (B)), and hyalorunidase (*hyl*), were selected for detection by PCR as described previously (Vankerckhoven et al., 2004; Hällgren et al., 2009).

*E. faecalis* ATCC29212, *E. faecium* E5 and *E. faecalis* E507 (Department of Medical Microbiology, Utrecht Medical Centre, UMC, The Netherlands) and *E. gallinarum* HSIEG1 (van den Bogert et al., 2013) (Laboratory of Microbiology, Wageningen University, The Netherlands) were used as positive controls for the detection of antibiotic resistance and virulence factor encoding genes. Amplicons were visualized by agarose gel electrophoresis.

### Clonal relatedness and analysis of population structure

In order to establish the clonal relationship of *Enterococcus* isolates, we applied the MLST schemes proposed by Ruiz-Garbajosa et al. (2006) and Homan et al. (2002) or *E. faecalis* and *E. faecium*, respectively.

Sequences were compared with published alleles, and sequence types (STs) were assigned using the MLST database (http://pubmlst.org/efaecium/ and http://pubmlst.org/efaecalis/). BAPS groups were determined as previously described (Willems et al., 2012). *E. faecalis* and *E. faecium* MLST products were stored in the MLST database (http://pubmlst.org/efaecium/ and http://pubmlst.org/efaecalis).

## Results

### Identification of intestinal *Enterococcus* isolates from ICU patients receiving SDD

**Figure 1:**
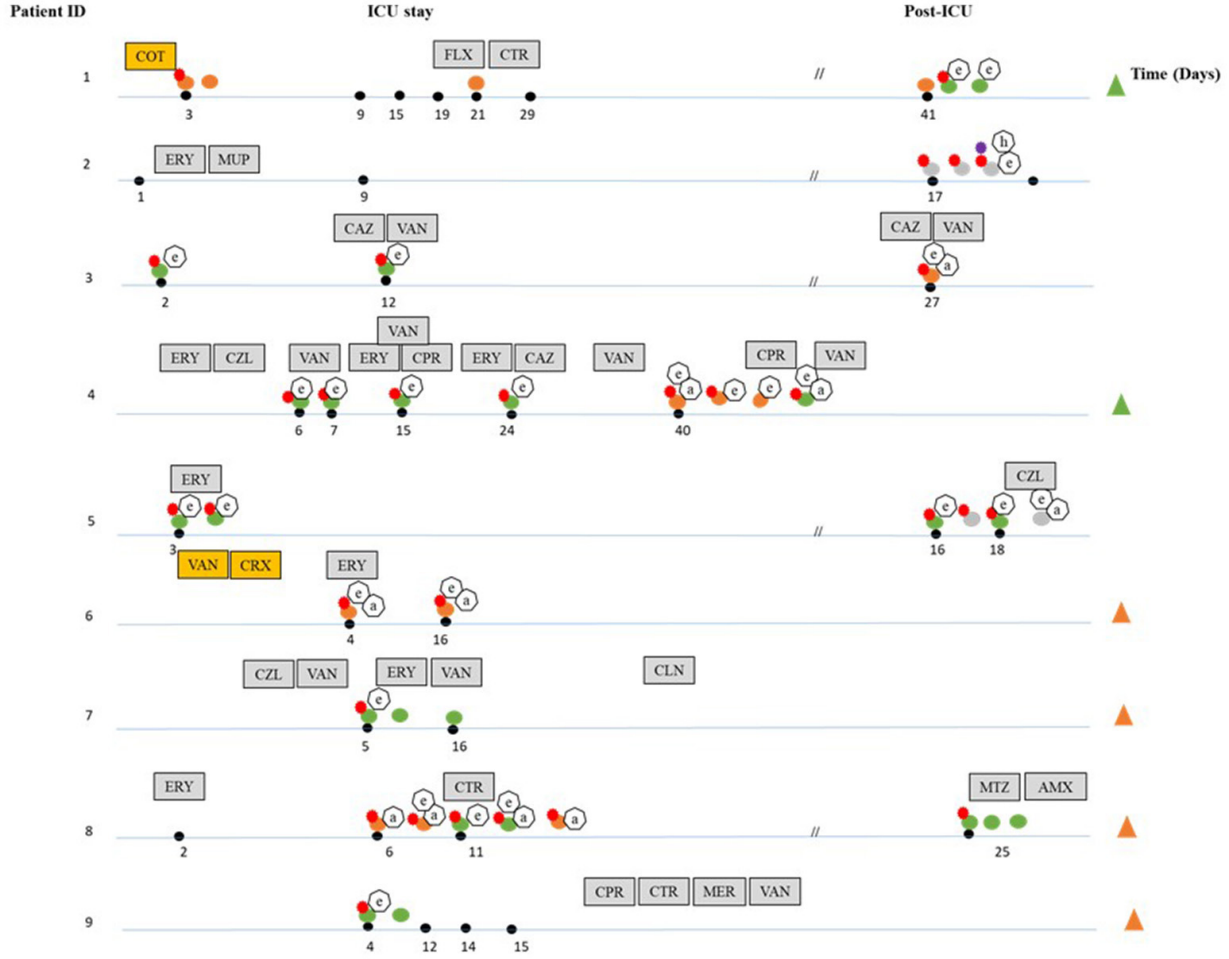
Overview of the dynamics of colonization by *Enterococcus* species and carriage of antibiotic resistance and virulence genes during and after ICU hospitalization. The black dots indicate days where fecal samples were taken during hospitalization. Discontinuation lines (//) indicate samples collected during the post-ICU period. The different species isolated are indicated by differently colored dots: orange (*E. faecalis*), green (*E. faecium*), dark gray (*E.sp*). Isolates not connected to a black dot were obtained from the sample closest to the left. The presence of antibiotic resistance genes is indicated by red (*erm*B) and purple dots (*van*C1). Virulence factors are shown in heptagonal shapes (a-*asa*1), (e-*esp-fm* and *esp-fs*), (h-*hyl*). Patients that developed nosocomial infections during ICU stay with *E. faecalis* and *E. faecium* are indicated by green (*E. faecium*) and orange (*E. faecalis*) triangles. Gray boxes indicate systemic antibiotics given under clinical indications at the specific time point indicated (ERY, erythromycin; VAN, vancomycin; CPR, ciprofloxacin; CTR, ceftriaxone; CAZ, ceftazidime; CZL, cefazoline; MER, meropenem; FLX, flucoxacilin; AMX, amoxacillin; CLN, clindamycin; MTZ, metronidazole; MUP, mupirosin), yellow boxes indicate systemic antibiotics given under clinical indications during the entire ICU stay (CRX, cefuroxime; COT, cotrimoxazole).

A total of nine ICU hospitalized patients that underwent SDD prophylactic antibiotic therapy were followed. From these patients, 34 fecal samples were collected. Besides SDD, additional antibiotics were administrated to seven patients for the control of infections and/or as an agent to accelerate gastric motility during ICU stay (Pilot, 1994; Galligan et al., 2005). Enterococci were isolated from 23 out of 34 fecal samples; the number of isolates per patient ranged from 2 (patient 6, 9) to 8 (patient 4, 8) (Table 1). Thirty six isolates were classified to the enterococcal species level by 16S rRNA gene sequencing and MLST products. The most commonly found species were *E. faecium* (23 isolates) and *E. faecalis* (13 isolates). The remaining five enterococcal isolates, all of which were isolated only during the post-ICU phase (Figure 1, Table 1), could not be unequivocally identified to the species level by 16S rRNA gene sequencing. Six patients receiving SDD developed nosocomial enterococcal infections during ICU stay (Figure 1), including one pleural infection caused by *E. faecium*, six urinary tract infections (two episodes in a single patient) caused by *E. faecalis* (five cases) and *E. faecium* (one case), and one central line catheter associated infection caused by *E. faecalis* (two episodes in a single patient). Unfortunately, however, the corresponding clinical isolates were not available for further analysis.

### Quantification of the enterococcal population

**Figure 2:**
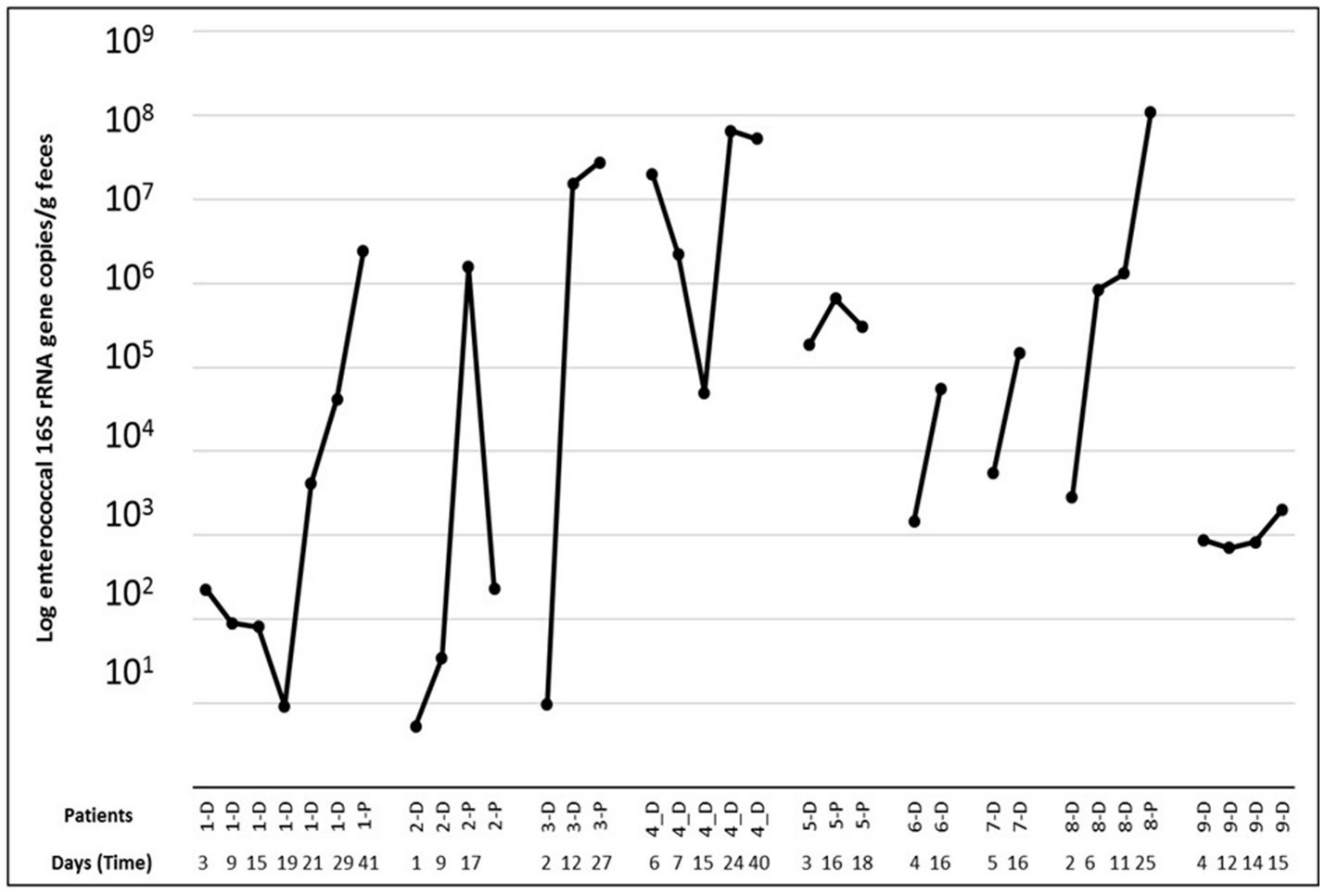
16S rRNA gene-targeted qPCR based quantification of the enterococcal population present per patient in the samples taken at different time points [during ICU (D) and Post-ICU (P)].

The quantification of the enterococcal population based on enterococcal 16S rRNA gene-targeted qPCR showed a significant increase in time from ICU stay to post ICU (6.0 × 10 ^6^ vs. 2.0 × 10 ^7^ Log DNA copies/g of feces; *p* < 0.05) in seven out of 9 patients. In the patients, substantial shifts in the absolute enterococcal 16S rRNA gene copy number were observed during the hospitalization period (Figure 2).

### Antimicrobial susceptibility

All isolates were vancomycin-susceptible (MIC 0.5–2 μg/ml), except for a single vancomycin resistant (MIC 16 μg/ml) isolate closely related to *E. gallinarum* (*E.sp*_3) (99% nucleotide identity).

Ampicillin resistance was detected in 25 out of 41 isolates, with the highest prevalence of resistant strains being found amongst *E. faecium* isolates[21 (91%) *E. faecium*, three (23%) *E. faecalis*, and one (20%) *E.sp* (*E.sp*_5)]. Resistance to tetracycline was detected in 19 out of 41 isolates [9 *E. faecalis*, seven *E. faecium*, and three *E. spp*. (*E.sp*_1, *E.sp*_2, *E.sp*_4)], the majority of which was obtained during ICU stay and in one patient during the first 72 h after admission.

### Detection of antibiotic resistance- and virulence factor-encoding genes

The presence of four of the genes that encode macrolide-lincosamide-streptogramin (MLS) resistance, namely *erm* (A), *erm* (B), *erm* (C), and *mef* (A)/*mef* (E) genes genes encoding the Major Facilitator Superfamily (MFS) transporter conferring resistance to erythromycin, were assayed by PCR-based detection. Our results revealed the presence of the *erm* (B) gene in 30 out of 41 enterococci isolates that were obtained during the entire study period. No other MLS_*B*_ resistance genes or MFS transporter genes were detected. From the group of vancomycin resistance genes tested, the *van* (C1) gene was identified in the single isolate that was also found vancomycin resistant (Figure 1, Table 1).

Three out of the four targeted genes encoding enterococcal virulence factors were detected. The *asa1* gene was frequently present in *E. faecalis* isolates (*n* = 7/13), whereas the *esp* gene was more often found in *E. faecium* isolates (*n* = 17/23). The *esp* gene was also present in two *E. sp*. isolates. Finally, the *hyl* gene was detected post-ICU in a single isolate of *E. faecium* and in a single isolate closely related to *E. gallinarum* (99% nucleotide identity). All isolates displayed alpha-haemolysis in blood agar. The *cyl* (B) gene, which would cause a beta-hemolytic phenotype (Semedo et al., 2003; Abriouel et al., 2008), was not detected in any of the isolates (Figure 1).

### Clonal relatedness and analysis of population structure

**Figure 3:**
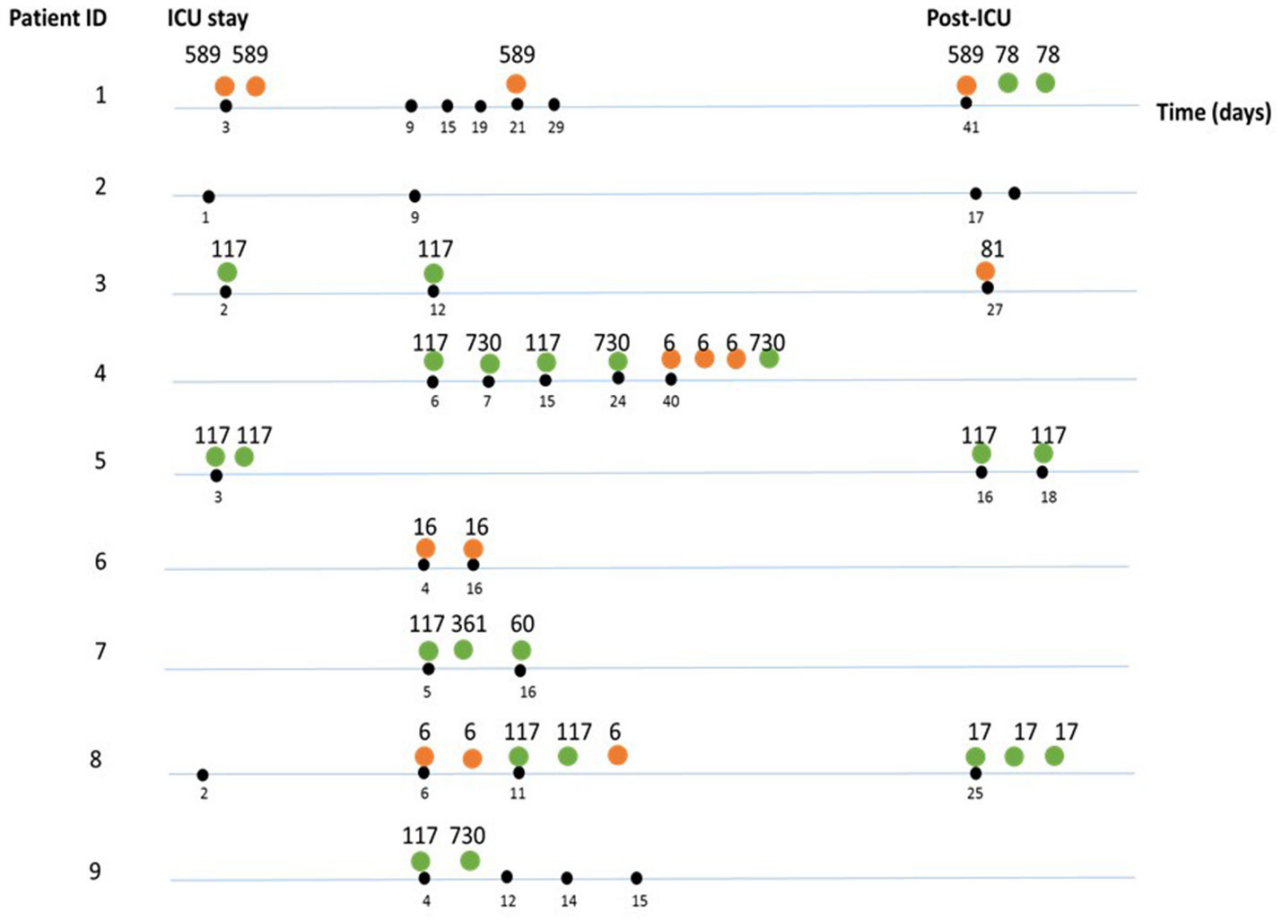
Sequence types (ST) identified per sample, per patient during and after SDD therapy. The differently colored dots indicate the species: orange (*E. faecalis*), green (*E. faecium*). Numbers indicate the sequence types. Black dots indicate the time point (days) where samples were taken during hospitalization. If multiple strains were isolated and characterized, these are shown to the right of each indicated timepoint.

Using MLST, we established the clonal relationship of all *E. faecium* and *E. faecalis* isolates obtained in this study. In total, we identified six different STs among the *E. faecium* isolates (Figure 3, Table 2). Further analysis of their population structure revealed that these STs belonged to three BAPS (sub) groups, which were previously associated with hospitalized patients (Willems et al., 2012). The majority of the STs belonged to BAPS group 2.1a (18 isolates), and 15 of them were resistant to ampicillin (ST117 *n* = 12, ST78 *n* = 2, and ST730 *n* = 1). Other sub-groups observed included BAPS 1.2 (2 isolates) as well as BAPS 3.3a2 (3 isolates). In four patients, we identified two or more different STs in the same patient during hospitalization (Figure 3).

**Table 2:** Sequence type (ST) and BAPS analysis of *Enterococcus faecalis* and *Enterococcus faecium* isolates.

	**Number of isolates**	**Sample collected (ICU-Post ICU**	**ST number**	**BAPS group**	**BAPS subgroup**
*E. faecalis* (*n* = 13)	6	ICU	6	1	
	2	ICU	16	1	
	4	3 ICU - 1 Post ICU	589	N.A^*^	
	1	Post ICU	81	3	
*E. faecium* (*n* = 23)	4	ICU	730	2	2.1a
	1	ICU	361	1	1.2
	1	ICU	60	1	1.2
	12	10 ICU—2 Post ICU	117	2	2.1a
	3	Post ICU	17	3	3.3a2
	2	Post ICU	78	2	2.1a

* *N.A: Not available*.

Among the *E. faecalis* isolates, we identified three STs (ST6, ST81, and ST16), which were previously detected among hospitalized patients (Willems et al., 2012), as well as a new ST (ST589), represented by four isolates (Figure 3, Table 2). All isolates belonging to ST589 were susceptible to ampicillin, and were obtained from a single patient from samples taken throughout the study. Three out of these four ST589 isolates carried the *ermB* gene.

From the group of *E. faecalis* isolates belonging to ST6 (*n* = 6), three carried *ermB, asa*, and *esp* genes and were susceptible to ampicillin, whereas the other three isolates displayed resistance to ampicillin. BAPS cluster analysis subdivided the *E. faecalis* isolates into BAPS groups 1 (eight isolates) and 3 (one isolate) (Table 2). In contrast to the situation in the *E. faecium* isolates, we neither detected the simultaneous presence of multiple *E. faecalis* STs nor clonal replacement over time within individual patients.

## Discussion

In the present study we characterized a group of *Enterococcus* species isolated from fecal samples of ICU patients receiving SDD therapy. We observed a pool of diverse enterococcal species, being *E. faecium* and *E. faecalis* the most prevalent species, both previously identified as important human pathogens associated with nosocomial infections (Cattaneo et al., 2010).

In three patients, these two species were detected in samples collected during the first 72 h, which could suggest that these patients were colonized with the recovered strains before ICU admission. This is in line with previous studies, as recently reviewed by Guzman Prieto and co-authors, showing that enterococci are present in healthy humans as well as in the environment, and that the abundance of resistance genes and mobile elements rapidly increases and facilitates colonization and subsequent infection in hospitalized patients (Guzman Prieto et al., 2016).

The clinical isolates causing nosocomial infections in the patients included in our study were not available for further analysis, which limited the possibility to clarify whether the infections derived from the isolates colonizing the patient in that period, and whether the isolates obtained from fecal material correspond to a nosocomial acquisition or selection of strains that were present in the gut microbiota of these patients prior to hospitalization. Other enterococcal isolates could not be identified to the species level (*n* = 5), although it should be noted that these were found only post-ICU. One possible explanation could be that due to the cessation of the SDD therapy, and thus aleviation of the corresponding antibiotic selective pressure during post-ICU stay, other species than *E. faecalis* and *E. faecium* were able to colonize the gut. From these isolates, three isolates were closely related to *E. gallinarum* and *E. avium* species. Both species have been identified in fecal samples of animals and healthy humans (Layton et al., 2010; Silva et al., 2011), and infrequently linked to human enterococcal infections (Tan et al., 2010; Varun et al., 2016). We cannot exclude that these other species were present in fecal samples taken at earlier time points at very low population size and thus evaded detection with the methods used in this study.

We were furthermore able to isolate more than one enterococcal species per sample in five out of nine patients. This highlights the importance of analyzing multiple colonies per culture to adequately sample the diversity of the enterococcal population. Moreover, qPCR analysis indicated an increase in the abundance of enterococci in seven out of nine patients. This shift could be due to the administration of antibiotics and to changes in the gut microbiota composition due to the antibiotic selective pressure. This is in line with previous studies based on qPCR analyses that showed that the enterococcal population increased in hospitalized patients receiving antibiotics compared to hospitalized patients without antibiotics and healthy volunteers (Bartosch et al., 2004).

Colonization by ampicillin-resistant *Enterococcus faecium* (ARE) is frequently associated with previous exposure to selective antibiotics, and ampicillin resistance is a specific trait for nosocomial isolates (de Regt et al., 2012).

In our study the highest prevalence of ampicillin resistance were found in *E. faecium* isolates compared to non-ampicillin resistant-*E. faecium* isolates. Similar results were reported by Ruiz-Garbajosa P., et al. (2012); indicating that this increased population most significantly contributes to the transmission and spread of enterococcal resistance in the ICU.

In our study, vancomycin resistance was not detected among *E. faecalis* and *E. faecium* isolates. This is in line with the previously reported prevalence (<1% for both *E. faecium* and *E. faecalis*) of vancomycin-resistance among enterococci in clinical infections in the Netherlands, as shown in the European Antimicrobial Resistance Surveillance System (EARSS) (ecdc.europa.eu/en/activities/surveillance/EARS-Net). The only vancomycin-resistant isolate was identified as being closely related to *E. gallinarum* (vancomycin MIC of 16 μg/ml), which carried the *van* (C1) gene that is naturally present in this species (Toye et al., 1997).

Resistance to tetracycline was detected in 46% of all 41 isolates (*n* = 19) and predominantly in *E. faecalis* isolates, which is in accordance with previous studies (Templer et al., 2008). Moreover, the presence of the macrolide resistance gene *erm* (B) was detected in 30 of these isolates (73%). We can not exclude, however, that other erythromycin resistance genes were present in these isolates. Although no vancomycin resistant *E. faecalis* and *E. faecium* populations were observed in the current study, our findings highlight the importance to perform a periodic surveillance during SDD therapy in ICU patients, in order to detect resistant *Enterococcus* spp. strains and prevent their dissemination as a preventive infection control measure.

Moreover, we observed that the pool of diverse enterococcal species identified in this study also harbored a variety of virulence genes that could contribute to infections in immunocompromised patients. The *esp* gene was the most prevalent virulence determinant detected throughout the study period followed by the *asa1* gene detected mainly during ICU stay, including two *E. faecium* and seven *E. faecalis* isolates, and one isolate closely related to *E. avium*. Similar results were previously reported (Billström et al., 2008; Hällgren et al., 2009; Sharifi et al., 2013). In addition, we detected the presence of the *hyl* gene in one *E. faecium* isolate and one isolate closely related to *E. gallinarum* only post-ICU. It should be noted, however, that the *hyl* gene has been identified not only in *E. faecium* and *E. faecalis*, but also in *E. casseliflavus, E. mundtii*, and *E. durans* isolated from food-stuffs (Trivedi et al., 2011), showing that the *hyl* gene can be present in a variety of *Enterococcus* spp. Furthermore, we cannot exclude that isolates obtained here contain other virulence genes that were not targeted in the present study.

Finally, the clonal relationship and population structure (BAPs groups) found in *E. faecium* and *E. faecalis* isolates indicated that the vast majority of our *E. faecium* isolates clustered in subgroups 2.1a and 3.3a2, representing separate hospital lineages that belong to clade A1 that contains most nosocomial *E. faecium* isolates (Willems et al., 2012). These BAPS groups are infrequent in healthy individuals (de Regt et al., 2012) suggesting that these isolates have been acquired during hospitalization.

Most *E. faecalis* isolates (62%) clustered in BAPS group 1, of which the majority belonged to ST 6 that was previously found in both hospitalized and non-hospitalized patients (Willems et al., 2012; Tedim et al., 2015).

In our study we observed the simultaneous presence of STs and clonal replacement over time among *E. faecium* isolates during ICU stay, whereas this was not the case for *E. faecalis*. Based on the current data, however, it is unclear whether the clonal replacement observed in *E. faecium* isolates was due to nosocomial strains or populations that were previously present in lower abundances. Future studies would be needed to test this hypothesis.

The prevalence of *Enterococcus* in ICU hospitalized patients, combined with the carriage of antibiotic resistance and virulence genes, described in this study, underlines the importance of this group of organisms as a potential cause of nosocomial infections in critically ill patients. Particular attention needs to be given to ICU patients during SDD therapy, with specific focus on the increased colonization by enterococci, even in non-endemic countries, considering that in SDD therapy, the enterococcal population is not a target group. SDD has been shown to not only decrease mortality and morbidity, but also to induce changes in the composition of the gut microbiota of patients (Benus et al., 2010; Buelow et al., 2014). Here we showed that it may increase the prevalence and extent of colonization by enterococci and provide insights into the diversity of the enterococcal strains that colonize these patients.

## Author contributions

TdJBG and PP: designed and performed the experiments, analyzed, and interpreted the data and wrote the paper. RW, JT and WvS: performed the MLST analysis and revised the work critically for intellectual content. HS and MWJvP: supervised the project, substantial contribution to revising it critically, and final approval of the version to be published.

### Conflict of interest statement

The authors declare that the research was conducted in the absence of any commercial or financial relationships that could be construed as a potential conflict of interest.
